# A Novel Role of Galectin-3 and Thyroglobulin in Prognosis and Differentiation of Different Stages of Thyroid Cancer and Elucidation of the Potential Contribution of Bcl-2, IL-8 and TNF-α

**DOI:** 10.3390/biomedicines10020352

**Published:** 2022-02-01

**Authors:** Tarek M. Okda, Gamal M. K. Atwa, Ahmed Fathy Eldehn, Naief Dahran, Khalaf F Alsharif, Ehab Kotb Elmahallawy

**Affiliations:** 1 Department of Biochemistry, Faculty of Pharmacy, Damanhour University, Damanhour 22511, Egypt; tarekokda@pharm.dmu.edu.eg; 2 Department of Biochemistry, Faculty of Pharmacy, Port Said University, Port Said 42515, Egypt; Gamal.Mohamed@pharm.psu.edu.eg; 3 Department of Otorhinolaryngology, Kasr Al-Ainy Medical School, Cairo University, Cairo 12613, Egypt; ahmedeldehn@kasralainy.edu.eg; 4 Department of Anatomy, Faculty of Medicine, University of Jeddah, Jeddah 21959, Saudi Arabia; ndahran@uj.edu.sa; 5 Department of Clinical Laboratory Sciences, College of Applied medical sciences, Taif University, P.O. Box 11099, Taif 21944, Saudi Arabia; alsharif@tu.edu.sa; 6 Department of Zoonoses, Faculty of Veterinary Medicine, Sohag University, Sohag 82524, Egypt

**Keywords:** thyroid cancer, thyroglobulin, interleukin-8, galectin-3, TNF-α

## Abstract

Thyroid cancer is among the most prevalent cancers with different types and stages. New markers are required for the prognosis and diagnosis of the disease. The present study aimed to detect the role of new markers, including galectin-3 (Gal-3) and thyroglobulin (TG), in the prognosis and staging of thyroid cancer. The study also investigated the potential apoptotic and inflammatory mechanisms involved in thyroid cancer through the determination of B-cell lymphoma 2 (Bcl-2), interleukin-8 (IL-8) and tumor necrosis factor α (TNFα) during the different stages of the cancer using a series of molecular methods. Histopathological and immunohistochemical examinations were also performed. A total of 300 subjects were classified into: 100 normal healthy subjects matched in age and sex, 100 patients with thyroid carcinoma stage I (T1N0M0) and 100 patients with thyroid carcinoma stage 2 (T2N1M1). Interestingly, the present study revealed a significant increase in the levels of TG and Gal-3 in thyroid cancer patients compared to the control group. Furthermore, the levels of Bcl-2, IL-8 and TNF-α significantly increased in the patient serum. The histopathological examination and immunohistochemical observations confirmed the molecular and hematological findings. Collectively, the present study concluded that serum TG and Gal-3 could be useful markers in the prognosis and staging of patients with thyroid cancer. Furthermore, the determination of Bax, Bcl-2, IL-8 and TNF-α levels constitute a major important marker for investigation of the mechanisms of apoptosis and inflammation in thyroid cancer. To our knowledge, this is the first study that used both galectin-3 and TG as tumor markers in the prognosis and differentiation between the different stages of cancer.

## 1. Introduction

Thyroid cancer remains among the most popular endocrine cancer worldwide and its incidence is continually increasing [1,2]. There are two types of thyroid cancer: differentiated and undifferentiated. The differentiated type includes papillary thyroid cancer (PTC), follicular thyroid cancer (FTC) and mixed carcinomas [3]. Among others, PTC represents more than 80% of thyroid gland cancers [4]. This global upsurge of thyroid cancer was explained by epidemiologists as a result of new diagnostic, screening and over-diagnosis or due to lifestyle changes such as obesity. Lacking of information and misunderstanding of the dedifferentiation is an index of a bad diagnosis for thyroid cancer; therefore, the incomplete characterization occurs due to its heterogeneity [5]. Clearly, evaluation of the environmental role and factors related to lifestyle seems very important in thyroid cancer patients. Taken into account, total thyroidectomy is the primary treatment for patients with thyroid cancer. Radioactive iodine (I-131) therapy after ablation therapy is also recommended in all patients. Generally, the prognosis and diagnosis of thyroid cancer are acceptable but there is still a small percentage of patients associated with poor prognostic parameters [6]. The protocol of primary diagnostic procedures for thyroid cancer is the examination of the thyroid gland by ultrasound and a fine-needle aspiration biopsy. The diagnosis depends on the clinical examinations, histopathology examination data and ultrasonographic signs [7].

It is noteworthy to state that both triiodothyronine (T3) and thyroxine (T4) are thyroid hormones, synthesized in the thyroid gland through a process that involves thyroglobulin (TG). Previous studies demonstrated a close connection between the low column of TG level to the low level of stimulated TG and the volume of thyroid tissue [8]. More importantly, TG was used as the initial tumor marker for patients with thyroid cancer; therefore, the serum TG level could be a prognostic marker to clinical management [9]. Furthermore, TG is considered one of a risk stratification system of response to the primary therapy besides being a valuable tool to follow-up of patients with thyroid cancer [10]. The relationship between the TG, thyroid stimulating hormone (TSH), the radioiodine ablation outcome and the clinical significance of TG doubling-time was also studied. Taken into consideration, angiogenesis and survival pathways for cancer stem cells have the ability to suppress the immune and provide growth factors [11]. Among others, nuclear factor kappa light chain enhancer of activated B cells (NF-κB) and interleukin-8 (IL-8) are markers of tumor progression in thyroid cancer. The inhibition of NF-κB signaling might decrease the thyroid cancer through different mechanisms including inhibition angiogenesis and proliferation of cancerous cells besides the reduction of VEGF and IL-8 [12,13]. It was also indicated that the expressions of pro-inflammatory factors, such as interleukin-1β (IL-1β) and tumor necrosis factor alpha (TNF-α), were increased in cancer and Alzheimer’s disease [14]. Furthermore, galectin-3 (Gal-3), a beta-galactoside binding protein, is involved in several biological functions and its expression increased in different types of cancer such as thyroid cancer. Moreover, Gal-8 is considered a diagnostic glycoprotein for liver diseases, and it was included in the mechanisms as apoptosis pathways by increasing the activity of caspase. Reviewing the available literature, little is known about the estimation of apoptotic and inflammatory markers and their potential influence on the various molecular mechanisms of the tumorgenesis and development of cancer. Given the above information, the present work was initially undertaken to assess the potential role of certain markers, including TG, Gal-3, Bax, Bcl-2, IL-8 and TNF-α, that could be useful in the prognosis, diagnosis and the differentiation of the different types of thyroid cancer.

## 2. Subjects and Methods

### 2.1. Ethical Approval and Informed Consent Statement

The present study was approved by the Research Ethical Committee, Faculty of Pharmacy, Damanhour University with ethical approval number 122PB24. The study was also performed in full accordance with the principle of Good Clinical Practice and according to the guidelines of the Helsinki Declaration and informed consent was obtained from all participants.

### 2.2. Sampling and Subject Criteria

The present study was conducted on samples collected from subjects who were admitted to the Damanhour Oncology Center, Egypt during the period between February 2018 and March 2020. This study was performed on 300 subjects classified into 3 groups: Group I (control group) which included 100 normal healthy subjects matched in age and sex; Group II (stage 1 group) that included 100 patients with thyroid carcinoma (T1N0M0) where the tumor was less than 2 cm, and there was no evidence of cancer in the regional lymph nodes and not spread to other parts of the body; and Group III (stage 2 group) that was composed of 100 patients with thyroid carcinoma (T2N1M1) where the tumor size was between 2–4 cm, and cancer had spread to the lymph nodes and other parts of the body. Demographics data were obtained from all participants and included age, chronic diseases and family history of thyroid cancer. The inclusion and exclusion criteria of the subjects were as follows:

Inclusion criteria: patients with thyroid cancer (papillary or follicular), stage I and II.

Exclusion criteria: patients with autoimmune diseases, other types of cancers, hepatic patients and kidney diseases were excluded from this study.

In accordance with the sample collection, about 5 mL of whole blood sample was collected from each participant after an overnight fast. For the complete blood count, 1 mL of the whole blood on EDTA was collected. The remaining blood samples (4 mL) were allowed to clot for 15 min at room temperature and centrifuged for 10 min at 10,000× *g* (14.810 g). The collected serum was stored at −20 °C for the assessment of IL-8, TG and TNF-α levels by the ELISA technique.

### 2.3. Complete Blood Count (CBC) Test

A full blood count (FBC) or CBC count was assessed by the ADVIA-2120 Haematology System (Bayer HealthCare, Diagnostics Division, Leverkusen, Germany) as described elsewhere [15].

### 2.4. Quantitative Real-Time PCR (qRT-PCR) of Bcl-2 and Bax

The total RNA was extracted from the serum of all samples by TRIzol^®^ (Invitrogen; Thermo Fisher Scientific, Inc, Waltham, MA, USA), according to the protocol of the manufacturer. The concentration and purity of RNA were determined by using a NanoDrop spectrophotometer (Thermo Fisher Scientific, Wilmington, USA). The assay system (Qiagen Strasse, Hilden, Germany) was used to estimate the mRNA expression of Bcl-2 (B-cell lymphoma 2) and Bax (Bcl-2-associated X protein). A volume of 1 µg RNA was reverse transcribed to form cDNA in the presence of the Master Mix (Thermo Fisher Scientific, Inc., Waltham, USA,). Gene expressions of Bcl-2 and Bax were measured by qRT-PCR using specific primers, which are shown in Table 1, through the following steps: (1) An initial 95 °C for 10 min, (2) denaturation at 95 °C for 15 sec repeated for 30 cycles, (3) annealing at 56 °C for 15 sec, (4) extension at 72 °C for 30 sec, 5) final extension at 72 °C for 10 min. The experiments were repeated three times. The relative mRNA expression of Bcl-2 and Bax compared to GAPDH were measured using the 2−ΔΔCq method [16].

### 2.5. Estimation of Serum TG Level

A volume of 100 µL samples or standards was added to the microtiter plate wells with a biotin-conjugated antibody specific to TG and incubated for 2 h at a temperature of 37 °C. The microtiter plate used was pre-coated with an antibody of TG. Later on, 100 μL of avidin conjugated to horseradish peroxidase was added to each microplate well and incubated for 1 h at 37 °C. Moreover, 100 μL of the substrates A and B mixture was added and incubated for 10 min at 37 °C to produce color. The intensity of the emitted light is directly proportional to the TG level in the sample or standard at 450 nm; the TG concentration (ng/mL) was calculated from the standard curve. A TG kit was purchased from HumaCLIA (1304 Langham Greek Dr, suite 226 Houston TX 77084 USA).

### 2.6. Estimation of Serum IL-8 Levels

A human IL-8 ELISA kit (cat. no.: IL831-K01; Eagle Biosciences, Inc. 20A Northwest Blvd., Suite 112, Nashua, NH, USA) was used for determination of serum IL-8 (pg/mL). This step involved a single-wash sandwich ELISA designed for the quantitative measurement of the IL-8 protein in human serum. Firstly, all reagents and working standards were prepared. A volume of 100 µL samples or standards was added to the microtiter plate wells and incubated for 1.5 h at 37 °C. Each well was aspirated and washed with wash buffer (350 µL); this step was repeated three times. A volume of 100 μl of the working solution of Biotin-conjugate was added to each well and incubated for 1 h at 37 °C. Wells were aspirated and washed with a wash buffer. The substrate solution (100 µL) was added to each well and incubated for 15 min at 37 °C. Then, the stop solution (100 µL) was added to each well. The optical density of each well was determined immediately using a microplate reader set to 450 nm.

### 2.7. Estimation of Serum TNF-α Level

The human TNF-α ELISA kit (cat. no.: TNA721-K43) was used for determination of serum TNF-α (pg/mL). It is a single-wash sandwich ELISA intended for the quantitative measurement of TNF-α in human serum. A volume of 100 µL samples, controls or standards and 50 µL of diluted biotinylated anti-TNF-α were added to the microtiter plate wells and incubated for 3 h at room temperature, in duplicate to the appropriate number of wells. Wells were aspirated and washed with the wash buffer. This later step was repeated three times. Streptavidin-HRP solution (100 μL) was added to each well and incubated for 30 min at room temperature. Wells were then aspirated and washed with the wash buffer. The TMB substrate solution (100 µL) was added to each well and incubated for 15 min at room temperature, then a stop solution (100 µL) was added to each well. The optical density of each well was determined immediately using a microplate reader set to 450 nm.

### 2.8. Estimation of Serum Gal-3 Levels

A human Gal-3 ELISA kit (Catalog number: GCT31-K01, Eagle Biosciences, Inc. 20A Northwest Blvd., Suite 112, Nashua, NH 03063, United States) was used in determination of serum Gal-3 (ng/mL). This assay is a quantitative sandwich ELISA. The immune plate was pre-coated with a polyclonal antibody specific for human Gal-3. Samples or standards (100 µL) were then added to the microtiter plate wells and incubated for 2 h at room temperature. Each well was aspirated and washed with a wash buffer and this step was repeated for a total of 3 washes. A detection antibody solution (100 μL) was added to each well and incubated for 1 h at room temperature. Wells were then aspirated and washed with a wash buffer. The STP-HRP solution (100 µL) was added to each well and incubated for 20 min at room temperature. Wells were aspirated and washed with wash buffer. A substrate solution (100 µL) was added to each well and incubated for 15 min at room temperature. Later on, a stop solution (100 µL) was added to each well. The optical density of each well was determined immediately using a microplate reader set to 450 nm and the serum Gal-3 concentration was determined from the standard curve.

### 2.9. Immunohistochemistry for Determination of TG in Thyroid Tissues

In this step, TG was detected immunohistochemically by using an antithyroglobulin antibody (code no.: EPR9730, Abcam, Cambridge Biomedical Campus, Cambridge, United Kingdom). The slides were washed for 5 min in the TBS buffer containing 0.025% Triton X-100 with gentle agitation, then blocked in 10% normal serum with 1% BSA in TBS for 2 h at room temperature and drained for a few seconds. The antigen was retrieved with a Tris-EDTA buffer commencing with the IHC staining protocol. Primary antibodies were diluted in TBS with 1% BSA and incubated for 10 h at 4 °C. The slides were rinsed 2 times for 5 min with TBS 0.025% Triton with gentle agitation and incubated in 0.3% H_2_O_2_ in TBS for 10 min. An enzyme-conjugated secondary antibody was applied to the slide, diluted in TBS with 1% BSA and incubated for 1 h at room temperature. Then, the slides were developed with chromogen for 10 min at room temperature and rinsed with running tap water for 5 min. The slides were then dehydrated, cleared and mounted.

### 2.10. Histopathological Analysis

A fine-needle aspiration biopsy from the thyroid gland was dissected and washed with 0.9% sterile saline solution. The liver was cut into small specimens and kept in 10% formalin solution. Formalin-fixed liver specimens were transferred to 70% ethanol and embedded in paraffin. Tissue sections were also stained with hematoxylin and eosin (HE) and examined under an optical microscope to detect pathological changes [17].

### 2.11. Statistical Methods

The statistical analysis was performed using the Graph Pad InStat Software Inc., program, version 4.0 Philadelphia, San Diego CA, USA (2003). Results were illustrated as mean ± SD and the significance was accepted with *p* < 0.05. A one-way ANOVA followed by the Tukey–Kramer test was used to perform the multiple comparisons.

## 3. Results

### 3.1. Demographical Data and Hematological Parameters

The demographical data of the subjects are shown in Table 2. All patients and control were selected from the same type of sex with a narrow range of age (control (42.27 ± 5.43); stage I (39.98 ± 7.18); stage II (43.88 ± 5.95)) and no history of thyroid cancer among patients’ families. Types and percentage of thyroid carcinoma were as follows: 77.4% papillary and 22.6% follicular among stage I patients while values were 81.2% papillary and 18.8% follicular among stage II group subjects.

In accordance with the CBC indices in experimental subjects, as shown in Table 3, the results showed a significant decrease in the hemoglobin concentration, white blood cell count and platelet count in stage I and II thyroid carcinoma patients compared with the control group. Meanwhile, there was a significant increase in the lymphocyte and neutrophil counts in the patients of stage I and II thyroid carcinoma compared with the control group.

### 3.2. The Gene Expressions of Bcl-2 and Bax

**Figure 1:**
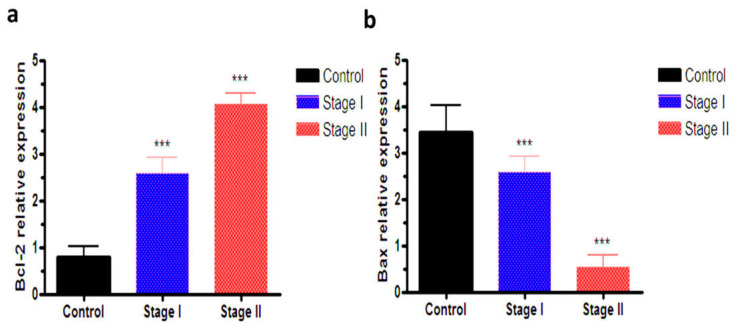
(**a**) The mRNA expression of Bcl2 in all studied groups; (**b)** Bax mRNA expression in all studied groups. Quantification analysis of mRNA was normalized, with Gapdh used as the housekeeping gene. Data are expressed as the mean ± SD. Significant difference from control group *** *p* < 0.001.

As shown in Figure 1a, there was a significant decrease in the gene expression of Bcl-2 in patients of stage I and II thyroid carcinoma compared with the control group. The gene expression of Bcl-2 in stage I and II groups were 2.78 and 3.92, respectively, compared to the control group. On the other hand, the gene expression of Bax was significantly increased in patients of stage I and II thyroid carcinoma compared with the control group. The gene expression of stage I and II groups were 0.53 and 2.60, respectively, compared to the control group’s 3.442 (Figure 1b).

### 3.3. Estimation of Serum TG, IL-8, TNF-α and Gal-3

**Figure 2:**
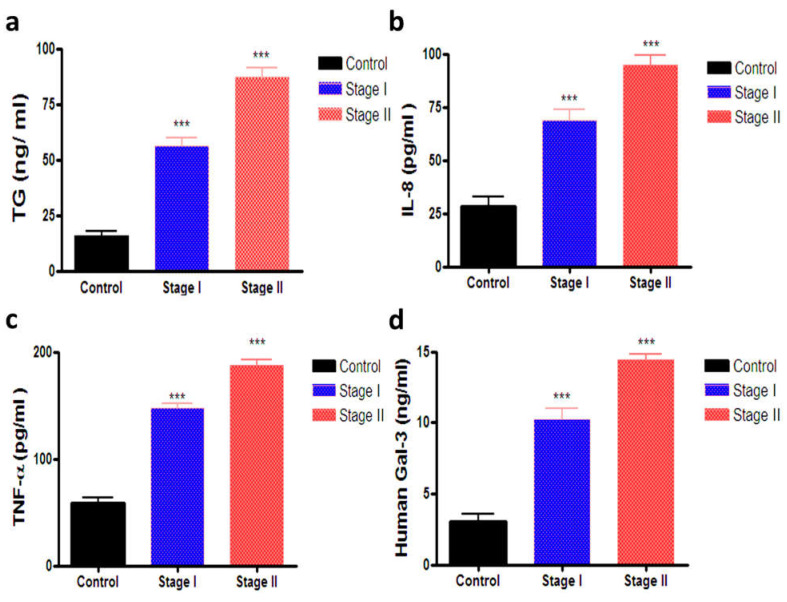
The serum levels of (**a**) TG, (**b**) IL-8, (**c**) TNF-α, (**d**) Gal-3 by using ELISA technique. Data wereexpressed as the mean ± SD. Data were analyzed using one-way ANOVA followed by Tukey post-hoc test at *p* < 0.05, Significant difference from the control group *** *p* < 0.001.

Table 4 represents the serum levels of TG, IL-8, TNF-α and Gal-3 levels. As shown in Table 4 and Figure 2a, there was a significant increase in the serum level of TG in stages I and II (56.19 ± 3.59 and 87.00 ± 4.20), respectively, compared with the control group (15.64 ± 2.11). In the same manner (Figure 2b), the serum level of IL-8 was significantly increased in stages I and II (68.72 ± 4.95 and 94.83 ± 4.32), respectively, compared with the control group (28.54 ± 4.46). Moreover, there was a significant increase in the serum level of TNF-α in stages I and II (147.8 ± 4.27 and 187.9 ± 4.74), respectively, versus the control group (58.41 ± 5.02) (Figure 2c). On the other hand, Figure 2d depicts that the serum level of Gal-3 was significantly increased in stage I and II (10.22 ± 0.81 and 14.46 ± 0.34), respectively, compared with the control group (3.04 ± 0.57).

### 3.4. Histopathological and Immunohistochemical Findings

**Figure 3:**
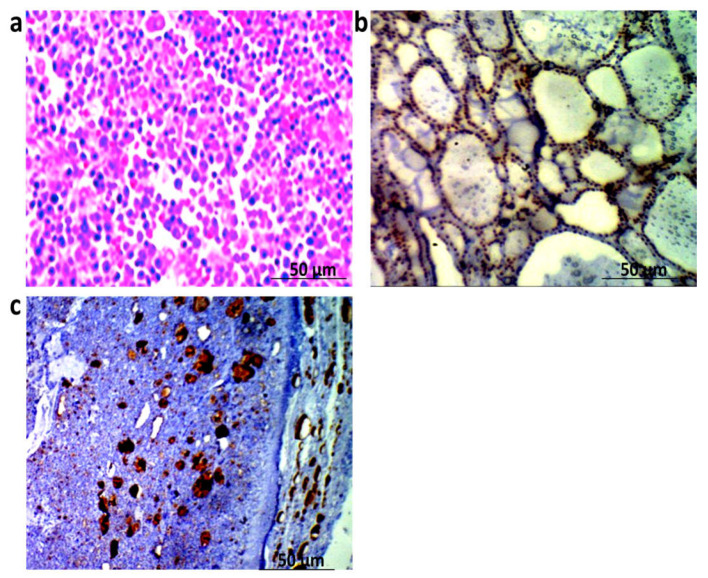
(**a**) Mild expression of TG in thyroid tissue of control group, TG antibody IHC, bar = 50 µm. (**b**) Thyroid gland tissue of stage I thyroid carcinoma group showing increase of TG expression within the proliferating nodule, TG antibody IHC, bar = 50 µm. (**c**) Thyroid gland tissue of stage II thyroid carcinoma group showing increase of TG expression within the proliferating nodule, TG antibody IHC, bar = 50 µm.

**Figure 4:**
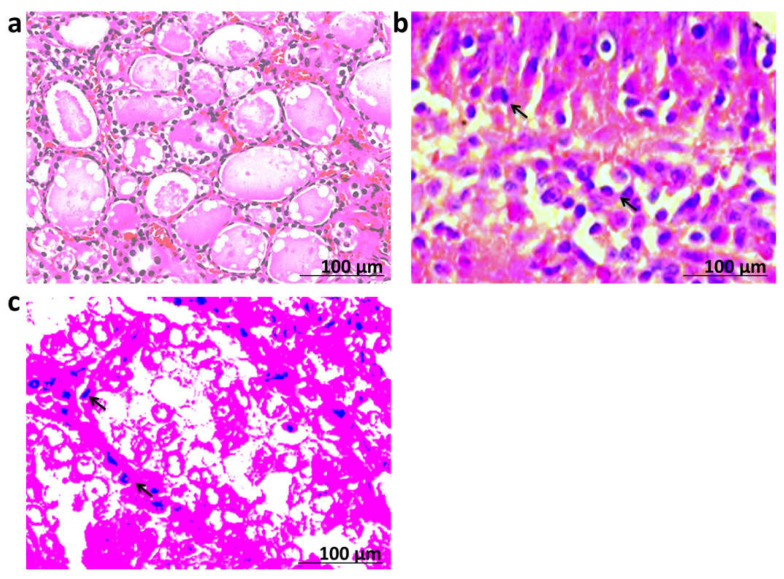
Histopathological changes in thyroid tissues; (**a**) control group showing normal architecture of thyroid cells. (**b**) Stage I group showing histopathological changes and heterogeneous in the thyroid cells (arrow). (**c**) Stage II group showing typical cytologic features and changes in nuclear features as nuclear size and shape and nuclear membrane irregularity (arrow). Slides were examined using H&E stain with magnification ×40 and scale bar = 100 µm.

The result showed the mild expression of TG within thyroid cells in the control group. Moreover, the expression of TG revealed a marked increase in stages I and II of thyroid patients which is shown in Figure 3. The histopathological characters of thyroid tissues obtained from the control group showed normal structures of thyroid cells. In addition, thyroid tissues obtained from stages I and II of thyroid patients showed histopathological changes and heterogeneous in the thyroid cells. As shown in Figure 4, there were typical cytologic features and changes in nuclear features such as nuclear size and shape and nuclear membrane irregularity (arrow).

## 4. Discussion

The periodical determination of new markers for the prognosis and diagnosis of thyroid cancer is mandatory. The present work provides a novel contribution about the molecular markers involved in the diagnosis and prognosis of thyroid cancer. Clearly, the study explored the various potential mechanistic pathways that might explain the tumorgenesis and development of cancer through the estimation of apoptotic and inflammatory markers such as Bcl-2, Bax, IL-8 and TNF-α. To the best of our knowledge, this is the first study that has combined the use of both TG and Gal-3 as markers in the prognosis and diagnosis of thyroid cancer. We focused the light on thyroid cancer and the cases were selected by matched age and sex to reduce the biological individual differences. As shown in our results, the TG levels were determined in all cases, and a significant increase in the TG level in both serum and tissue was observed in stage II and stage I groups compared to the control group. The results of immunohistochemistry confirmed a significant increase in TG expressions at the tissue level in stage I and stage II patients compared to the control group. This finding is consistent with a study conducted by Park et al. 2018 [18], who found that the levels of serum TG and the ratio of TG/TSH significantly increased in patients with thyroid cancer with and without recurrences. This reflects the hypothesis that TG levels maybe have a predicting value in recurrent thyroid cancer.

Several studies have shown the use of Gal-3 as a biomarker for thyroid cancer besides being a good target for the molecular imaging of thyroid cancer. Gal-3 has an important role in the tumor process and progression. Currently, Gal-3 targeting is under examination for its possible utility as a therapy for thyroid cancer [19]. Our study also revealed that the level of Gal-3 was estimated in the serum for all cases. Gal-3 is one of these proteins markers that produced a significant difference. In this respect, there was a significant increase in the expression of Gal-3 in thyroid cancer in both stages I and stage II, but the increase was higher in stage II patients. The significant elevation of serum Gal-3 agrees with the study conducted by Makki et al. 2013 [20] in which serum levels of about five protein markers were increased in thyroid masses’ patients compared to normal individuals. Meanwhile, the difference between the PTC and benign was not considerable. To our knowledge, no previous study explored the combined use of TG and Gal-3 as molecular markers in the prognosis and diagnosis of this type of cancer.

The Bax protein is an apoptotic agent present in the cytoplasm with a similar structure to the Bcl-2 structure; therefore, it is an antagonist protein for Bcl-2. It should be stressed that Bax proteins stimulate the release of cytochrome-C from mitochondria which is then transferred to the cytoplasm, combined with the Apaf-1 protein, and then activates the mitochondrial caspase (caspase-9) responsible for apoptosis. Furthermore, Bax is a Bcl-2-associated X protein which is an apoptotic activator besides its role in stimulation of the release of cytochrome c and caspase-3. Clearly, the gene expression of Bax was determined in the present work to investigate the mechanisms and stages of thyroid cancer development. Revising the available literature, several studies reported that the expression of some inhibitors of Bcl-2 cell death was reduced in thyroid cancer patients, while inducers of Bax cell death increased in those patients [21,22]. These previous findings are in agreement with our results that showed the decrease in the serum level of Bax in both stages I and II thyroid cancer patients. Regarding the gene expression of Bcl-2, which plays an important role in regulating the survival of the cell and acts as an anti-apoptotic agent, our study estimated its level in all study subjects and the work revealed an increase in the serum level of Bcl-2 in thyroid cancer patients in both stages I and II. This finding is consistent with a study conducted by Jing et al. 2021 [23] who reported that the expression of Bcl-2 appears to show a significant decrease in cancer. A previous study hypothesized that the anticancer activity of natural products might involve the enhancement of apoptotic markers [24]. Moreover, another study reported that the Bcl-2 as an apoptotic marker was increased with the incidence of cancer [25], which is consistent with the present results.

It should be noted that interleukins are inflammatory molecules in the microenvironment of cancer. Among others, IL-8 is a potent chemokine synthesized by macrophages and stored by endothelial cells in vesicles. Different types of tumors also produce this chemokine, which in this context exerts different functions and its concentration correlates with tumor severity [26]. Moreover, IL-8 and Toll-like receptors (TLRs) are playing a vital role in host defense against inflammation and cancer [27]. Furthermore, IL-8 is a downstream regulator of nuclear factor kappa light chain enhancer of activated B cells NF-κB pathways in thyroid cancer progression [28]. It is therefore not surprising to state that the IL-8 serum level is a useful marker as a pharmacodynamic marker to detect the early response of immunotherapy [29]. As shown in our work, there was a significant increase in the serum level of IL-8 in both stage I and stage II patients. IL-8 is a member of the neutrophil-specific chemokine beyond its role as a potent activating and chemotactic factor. The present findings are supported by Chuang et al. 2020 [30], who indicated that inflammation is important to the formation and progression of cancers. Interleukins are inflammatory molecules in the microenvironment of cancer. IL-8 also promotes the migration and proliferation of thyroid cancer cells. Moreover, interleukins were used in the treatment and prognosis of thyroid cancer. It is noteworthy to state that TNF-α (cachectin) is a potent cytokine synthesized and primarily produced by macrophages, monocytes, neutrophils and lymphocytes and serves many critical functions in cell signaling and is implicated in the inflammatory response [31]. In addition, this protein has a major role as a regulator of immune response. As shown in our present results, the level of TNF-α increased in the serum of both stage I and stage II patients compared to the control group. This finding is consistent with a study conducted by Zhang et al. [32], who reported that the expression of mRNA and the protein of serum TNF-α in thyroid cancer patients were higher than those of the control group. It seems that the level of TNF-α was decreased by the surgical resection of tissue. Collectively, the present results reflect that TNF-α could be used as a prognostic and diagnostic marker of Hashimoto’s disease in patients with thyroid cancer.

Histopathological examination and immunohistochemical methods are considered important tools for the investigation of the novel biomarkers in personalized medicine [33,34]. In the present work, our study depicted a marked increase in the expression of tissue TG in stages I and II of thyroid patients versus the control group. Furthermore, a series of histopathological changes and heterogeneous in the thyroid cells were observed in thyroid tissues obtained from stages I and II of the thyroid groups. Likewise, typical cytological features and changes in nuclear features, including nuclear size and shape and nuclear membrane irregularity, were observed in stages I and II of the thyroid patients. These results are in agreement with a previous study which revealed that TG was positive in all patients with well-differentiated thyroid cancer (WDTC) [9]. The present findings also reflect that tissue TG and TTF1 could be accurate diagnostic factors in thyroid carcinoma. However, it should be borne in mind that the periodical check of the specificity and sensitivity of these methods should be practiced, together with the careful interpretation of the results [35,36].

## 5. Conclusions

Given the above information, the present study concluded novel data about the prognostic and diagnostic value of various molecular markers in patients with thyroid cancer. In addition to their prognostic values, serum TG and Gal-3 were used as close markers to determine the stage of cancer in thyroid cancer patients. The present data also reveal that the determination of Bax, Bcl-2, IL-8 and TNF-α levels might possess a potential prognostic and diagnostic value in thyroid cancer patients, reflecting that apoptosis and inflammation are important mechanisms in thyroid cancer. These molecular findings were also supported by a series of histopathological and immunohistochemical findings that concluded that tissue TG and TTF1 could be accurate diagnostic factors in patients with thyroid carcinoma. Further future research is warranted to explore more about the potential molecular apoptotic and inflammatory pathways involved in thyroid cancer patients.

## Figures and Tables

**Table 1:** Details of NCBI reference sequence of the three sets of primers of Bcl-2, Bax and Gapdh.

Primer	Sequence (5′-3′) Direction
Bcl-2	F: 5′CATCGCCCTGTGGATGACTG 3′
	R: 5′GGCCATATAGTTCCACAAAGGC 3′
Bax	F: 5′GTCTCCGGCGAATTGGAGAT 3′
	R: 5′ACCCGGAAGAAGACCTCTCG 3′
Gapdh	F: 5′ GTATCGGACGCCTGGTTAC 3′
	R: 5′ CTTGCCGTGGGTAGAGTCAT 3′

**Table 2:** Data and clinical characteristics of all subjects in the study.

Data	Control	Stage 1	Stage II
Sex	Male	Male	Male
Age	42.27 ± 5.43	39.98 ± 7.18	43.88 ± 5.95
History of thyroid cancer	None	None	None
Types of thyroid carcinoma	-------	77.4% Papillary 22.6% Follicular	81.2% Papillary 18.8% Follicular

**Table 3:** Mean ± SD of CBC indices in all studied groups (control, stage I and stage II).

Biochemical Indices	Control	Stage 1	Stage II
Hb (g/dl)	12.28 ± 0.90	11.32 ± 0.63 *	11.01 ± 0.92 *
RBCs × 10^6^	5.79 ± 1.23	4.67 ± 2.03 *	4.09 ± 1.93 *
Hct	39.91 ± 4.53	37.81 ± 3.82	33.22 ± 2.77 *
Platelets × 10^3^	248.55 ± 16.97	207.21 ± 21.17 *	201.90 ± 17.51 *
WBCs × 10^3^	7.05 ± 2.25	5.82 ± 1.87 *	5.03 ± 1.33 *
Lymphocytes	29.81 ± 3.85	37.28 ± 3.17 *	43.75 ± 4.55 *
Neutrophils	34.55 ± 4.09	49.85 ± 3.33 *	47.18 ± 3.08 *

Hb: hemoglobin, RBCs: red blood cells, Hct: hematocrit, WBCs: white blood cells. * Significance compared to the control group *p* < 0.001.

**Table 4:** Mean ± SD of serum TG, IL-8, TNF-α and Gal-3 levels by ELISA technique.

Biochemical Indices	Control	Stage 1	Stage II
TG (ng/mL)	15.64 ± 2.11	56.19 ± 3.59 *	87.00 ± 4.20 *
IL-8 (pg/mL)	28.54 ± 4.46	68.72 ± 4.95 *	94.83 ± 4.32 *
TNF-α (pg/mL)	58.41 ± 5.02	147.8 ± 4.27 *	187.9 ± 4.74 *
Gal-3 (ng/mL)	3.04 ± 0.57	10.22 ± 0.81 *	14.46 ± 0.3378 *

TG: thyroglobulin, IL-8: interleukin 8, TNF-α: tumor necrosis factor-α, Gal-3: galectin-3. Data were represented as the mean ± SD.* Significance compared to the control group *p* < 0.05.

## Data Availability

The data that support the findings of this study are contained within the article. More information is available on request from the corresponding author.

## References

[1] Siegel R.L., Miller K.D., Jemal A. (2019). Cancer statistics, 2019. CA Cancer J. Clin..

[2] Bray F., Ferlay J., Soerjomataram I., Siegel R.L., Torre L.A., Jemal A. (2018). Global cancer statistics 2018: GLOBOCAN estimates of incidence and mortality worldwide for 36 cancers in 185 countries. CA Cancer J. Clin..

[3] Xing M. (2013). Molecular pathogenesis and mechanisms of thyroid cancer. Nat. Rev. Cancer.

[4] Fagin J.A., Wells S.A. (2016). Biologic and clinical perspectives on thyroid cancer. N. Engl. J. Med..

[5] Luo H., Xia X., Kim G.D., Liu Y., Xue Z., Zhang L., Shu Y., Yang T., Chen Y., Zhang S. (2021). Characterizing dedifferentiation of thyroid cancer by integrated analysis. Sci. Adv..

[6] Gillanders S., O’Neill J. (2018). Prognostic markers in well differentiated papillary and follicular thyroid cancer (WDTC). Eur. J. Surg. Oncol..

[7] Mamedov U., Khodjaeva D. (2021). Modern Diagnostic Approachкetreatment of Thyroid Cancer. Int. J. Dev. Public Policy.

[8] Spencer C.A., LoPresti J.S. (2008). Technology Insight: Measuring thyroglobulin and thyroglobulin autoantibody in patients with differentiated thyroid cancer. Nat. Clin. Pract. Endocrinol. Metab..

[9] Zahra H.O., Omran G.A., Gewely A.G., Eldehn A.F., Abdo W., Elmahallawy E.K., Okda T.M. (2021). Prognostic Value of Serum Thyroglobulin and Anti-Thyroglobulin Antibody in Thyroid Carcinoma Patients following Thyroidectomy. Diagnostics.

[10] Prpić M., Franceschi M., Romić M., Jukić T., Kusić Z. (2018). Thyroglobulin as a Tumor Marker in Differentiated Thyroid Cancer–Clinical Considerations. Acta Clin. Croat..

[11] Eyler C.E., Rich J.N. (2008). Survival of the fittest: Cancer stem cells in therapeutic resistance and angiogenesis. J. Clin. Oncol..

[12] Bauerle K.T., Schweppe R.E., Haugen B.R. (2010). Inhibition of nuclear factor-kappa B differentially affects thyroid cancer cell growth, apoptosis, and invasion. Mol. Cancer.

[13] Haibe Y., Kreidieh M., El Hajj H., Khalifeh I., Mukherji D., Temraz S., Shamseddine A. (2020). Resistance Mechanisms to Anti-angiogenic Therapies in Cancer. Front. Oncol..

[14] Ali A.A., Khalil M.G., Abd El-latif D.M., Okda T., Abdelaziz A.I., Kamal M.M., Wahid A. (2022). The influence of vinpocetine alone or in combination with Epigallocatechin-3-gallate, Coenzyme COQ10, Vitamin E and Selenium as a potential neuroprotective combination against aluminium-induced Alzheimer’s disease in Wistar Albino Rats. Arch. Gerontol. Geriatr..

[15] Harris N., Kunicka J., Kratz A. (2005). The ADVIA 2120 hematology system: Flow cytometry-based analysis of blood and body fluids in the routine hematology laboratory. Lab. Hematol..

[16] Livak K.J., Schmittgen T.D. (2001). Analysis of relative gene expression data using real-time quantitative PCR and the 2− ΔΔCT method. Methods.

[17] Hahn H.P., Bundock E.A., Hornick J.L. (2006). Immunohistochemical staining for claudin-1 can help distinguish meningiomas from histologic mimics. Am. J. Clin. Pathol..

[18] Park S., Jeon M.J., Oh H.-S., Lee Y.-M., Sung T.-Y., Han M., Han J.M., Kim T.Y., Chung K.-W., Kim W.B. (2018). Changes in serum thyroglobulin levels after lobectomy in patients with low-risk papillary thyroid cancer. Thyroid.

[19] Li J., Vasilyeva E., Wiseman S.M. (2019). Beyond immunohistochemistry and immunocytochemistry: A current perspective on galectin-3 and thyroid cancer. Expert Rev. Anticancer. Ther..

[20] Makki F.M., Taylor S.M., Shahnavaz A., Leslie A., Gallant J., Douglas S., Teh E., Trites J., Bullock M., Inglis K. (2013). Serum biomarkers of papillary thyroid cancer. J. Otolaryngol.-Head Neck Surg..

[21] Hamivand Z., Haddadi G., Fardid R. (2018). Expression of Bax and Bcl2 genes in peripheral blood lymphocytes of patients with differentiated thyroid cancer. J. Med. Phys..

[22] Spanos S., Rice S., Karagiannis P., Taylor D., Becker D., Winston R., Hardy K. (2002). Caspase activity and expression of cell death genes during development of human preimplantation embryos. Reproduction.

[23] Jing C., Li Y., Gao Z., Wang R. (2022). Antitumor activity of Koningic acid in thyroid cancer by inhibiting cellular glycolysis. Endocrine.

[24] Okda T.M., Abd-Εlghaffar S.K., Katary M.A., Abd-Alhaseeb M.M. (2021). Chemopreventive and anticancer activities of indomethacin and vitamin D combination on colorectal cancer induced by 1,2-dimethylhydrazine in rats. Biomed. Rep..

[25] Okda T.M., Katry M.A., Ragab N.M., Shalkami A.-G.S. (2021). Phytic acid potentiates oxaliplatin effects in colorectal cancer induced by 1, 2-DMH: The role of miR-224 and miR-200a. Contemp. Oncol..

[26] Gonzalez-Aparicio M., Alfaro C. (2019). Influence of Interleukin-8 and Neutrophil Extracellular Trap (NET) Formation in the Tumor Microenvironment: Is There a Pathogenic Role?. J. Immunol. Res..

[27] Fakhry N., Gowily A., Okda T., Houssen M. (2020). Serum soluble toll-like receptor 2 and 4 as diagnostic and prognostic biomarkers for non-Hodgkin lymphoma. Contemp. Oncol..

[28] Bauerle K.T., Schweppe R.E., Lund G., Kotnis G., Deep G., Agarwal R., Pozdeyev N., Wood W.M., Haugen B.R. (2014). Nuclear Factor κB–Dependent Regulation of Angiogenesis, and Metastasis in an In Vivo Model of Thyroid Cancer Is Associated With Secreted Interleukin-8. J. Clin. Endocrinol. Metab..

[29] Alfaro C., Sanmamed M.F., Rodríguez-Ruiz M.E., Teijeira Á., Oñate C., González Á., Ponz M., Schalper K.A., Pérez-Gracia J.L., Melero I. (2017). Interleukin-8 in cancer pathogenesis, treatment and follow-up. Cancer Treat. Rev..

[30] Xi C., Zhang G.-Q., Sun Z.-K., Song H.-J., Shen C.-T., Chen X.-Y., Sun J.-W., Qiu Z.-L., Luo Q.-Y. (2020). Interleukins in thyroid cancer: From basic researches to applications in clinical practice. Front. Immunol..

[31] Bär E., Whitney P.G., Moor K., Sousa C.R.e., LeibundGut-Landmann S. (2014). IL-17 regulates systemic fungal immunity by controlling the functional competence of NK cells. Immunity.

[32] Zhang N., Wang Q., Tian Y., Xiong S., Li G., Xu L. (2019). Expressions of IL-17 and TNF-α in patients with Hashimoto’s disease combined with thyroid cancer before and after surgery and their relationship with prognosis. Clin. Transl. Oncol..

[33] Carneiro G., Radcenco A.L., Evaristo J., Monnerat G. (2019). Novel strategies for clinical investigation and biomarker discovery: A guide to applied metabolomics. Horm. Mol. Biol. Clin. Investig..

[34] Dunstan R.W., Wharton K.A., Quigley C., Lowe A. (2011). The Use of Immunohistochemistry for Biomarker Assessment—Can It Compete with Other Technologies?. Toxicol. Pathol..

[35] Hawes D., Shi S.-R., Dabbs D.J., Taylor C.R., Cote R.J. (2009). Immunohistochemistry. Mod. Surg. Pathol..

[36] Kim S.-W., Roh J., Park C.-S. (2016). Immunohistochemistry for Pathologists: Protocols, Pitfalls, and Tips. J. Pathol. Transl. Med..

